# Bells, bomas and beefsteak: complex patterns of human-predator conflict at the wildlife-agropastoral interface in Zimbabwe

**DOI:** 10.7717/peerj.2898

**Published:** 2017-01-24

**Authors:** Andrew J. Loveridge, Timothy Kuiper, Roger H. Parry, Lovemore Sibanda, Jane Hunt Hunt, Brent Stapelkamp, Lovelater Sebele, David W. Macdonald

**Affiliations:** 1 WildCRU, Zoology Department, University of Oxford, Oxford, United Kingdom; 2 Victoria Falls Wildlife Trust, Victoria Falls, Zimbabwe; 3 Hwange Main Camp, Zimbabwe Parks and Wildlife Management Authority, Hwange National Park, Zimbabwe

**Keywords:** Lion, Hyaena, Leopard, Human-wildlife-conflict, Livestock, Predation, Husbandry, Boma

## Abstract

Reports of livestock depredation by large predators were systematically collected at three study sites in northwestern Zimbabwe from 2008–2013. We recorded 1,527 incidents (2,039 animals killed and 306 injured). Lions (*Panthera leo*) and spotted hyaenas (*Crocuta crocuta*) were mostly responsible, and cattle and donkeys most frequently attacked. Patterns of predation were variable among study sites. Nevertheless, some overall patterns were apparent. Predators selected livestock close to the size of their preferred wild prey, suggesting behaviours evolved to optimise foraging success may determine the domestic species primarily preyed upon. Most attacks occurred when livestock were roaming outside and away from their ‘home’ protective enclosures at night. Hyaena attacks were largely nocturnal; lions and leopards (*Panthera pardus*) were more flexible, with attacks occurring by day and at night. Livestock fitted with bells suffered a disproportionate number of attacks; the sound of bells appears to have conditioned predators to associate the sound with foraging opportunities. Lion and hyaena attacks on cattle were more frequent in the wet season suggesting that seasonal herding practices may result in cattle vulnerability. Only a small proportion of conflict incidents were reported to wildlife management officials with a bias towards lion predation events, potentially prejudicing conflict management policies. Predation on domestic stock involves an intricate interplay between predator behaviour and ecology on the one hand and human behaviour and husbandry practices on the other. Our data suggest that improved livestock husbandry (supervision of grazing animals, protection at night in strong enclosures) would greatly reduce livestock depredation.

## Introduction

The threats facing the world’s largest carnivores (habitat loss and fragmentation, conflict with humans over livestock, prey depletion, etc.) often act in concert and emerge from a complex interplay of ecological and sociological factors (Macdonald, Loveridge & Rabinowitz, 2010). A sound scientific understanding of this complexity is indispensable to effective carnivore conservation. Over and above their contribution to ecosystem resilience as apex predators (Ripple et al., 2014), large carnivores are intimately interwoven with many human cultures (Kellert et al., 1996), they acts as umbrella species for wider habitat protection (Sergio et al., 2005), and they are charismatic flagships for broader conservation issues (Macdonald et al., 2015). Their effective conservation is therefore paramount. In this paper we present a detailed analysis of a long-term dataset of carnivore attacks on livestock in Zimbabwe with the aim of contributing to the evidence base for a better understanding of human-predator conflict and its effective mitigation.

Conflict with people over livestock has resulted in the extirpation of many carnivore species from much of their historical range in many parts of the world (Frank & Woodroffe, 2001; Loveridge et al., 2010b). In Africa, European colonists eliminated most large carnivores from agricultural land in the late 19th and early 20th Centuries (Woodroffe, 2000), though many predator populations continue to exist in regions of low human population density and in extensive protected area networks. Nevertheless, the human population in sub-Saharan Africa is expected to double from 1.2 to nearly 2.5 billion in the next 50 years (United Nations, 2015). This expansion, together with rising global demand for food, will inevitably lead to the expansion of human land-use, thereby intensifying conflict between people and carnivores at the wildlife-agriculture interface.

Large predators in African savannah ecosystems are almost all under threat (Ripple et al., 2014), and this is true of the large carnivores in our study system of northwestern Zimbabwe. African lions (*Panthera leo*) have declined continent wide and are listed as ‘Vulnerable’ (Bauer et al., 2015). Conflict with people accounts for a significant proportion of lion mortality in our study population (Loveridge et al., 2010a). African leopards (*P. pardus*) are listed as ‘Near Threatened’ by the IUCN and Appendix I by CITES (Henschel et al., 2008), while Spotted hyaena (*Crocuta crocuta*) suffer extensive persecution on agricultural land (Bohm & Höner, 2015). Cheetah (*Acinonyx jubatus*) and African wild dog (*Lycaon pictus*) are both endangered and have suffered extreme range collapse (Durant et al., 2015; Woodroffe & Sillero-Zubiri, 2012).

While human-wildlife conflict is a commonly documented phenomenon across sub-Saharan Africa (Koziarski, Kissui & Kiffner, 2016; Loveridge et al., 2016; Rust, 2016), effective monitoring of conflict incidents is uncommon. Consequently, our understanding of the patterns and drivers of conflict is frequently poor. Furthermore, across African savannah systems, there is considerable variation in predator guild structure, human socio-cultural practices, climate, and ecosystem structure. These factors make the interpretation of interactions between humans and wildlife complex, with broad generalisations proving elusive. Examples of factors affecting the intensity of conflict include: previous exposure of communities to conflict, levels of wealth and education, ethnicity, livestock ownership patterns, husbandry practices, political integration, historical grievances and the presence, abundance and behavioural ecology of different predators in the system (Dickman, 2010; Hazzah, Borgerhoff-Mulder & Frank, 2006; Inskip & Zimmermann, 2009). Given these complexities, any single approach to conflict mitigation is unlikely to be universally applicable, and successful mitigation strategies focused on particular predator species or localised problems are likely to fail if transferred to other situations or contexts. Whilst studies of human-predator conflict over livestock depredation almost universally indicate that improvements to livestock protection result in lower levels of predation (Mazzoli, Graipel & Dunstone, 2002; Woodroffe et al., 2007), appropriate solutions still need to be tailored to site specific circumstances.

In this study, we examine the complexity of human-predator conflict in three separate rural communities adjacent to National Parks in northwestern Zimbabwe (these communal areas and Parks fall within the broader transboundary Kavango-Zambezi Transfrontier Conservation Area). Here we define complexity as the linked ecological (specifically predator behaviour) and sociological (specifically livestock husbandry) factors that explain how intensely, when, and why predators attack livestock. Using a five-year dataset of over 1,500 separate carnivore attacks on livestock, together with current and previously published data on husbandry practices, we aim to disentangle some of this complexity by investigating (and comparing among sites) the following:

(1) Predator behaviour: identifying the predator species involved and differences in their overall impact, preference for different livestock species, diel and seasonal variations in their attack frequency, and their responses to husbandry practices.
(2) Husbandry practices: analysing the influence of certain husbandry practices (the use of protective enclosures, bells fitted to livestock and seasonal herding practices) on the patterns and intensity of attacks (linking this back to predator behaviour).

## Methods

### Study area

**Figure 1:**
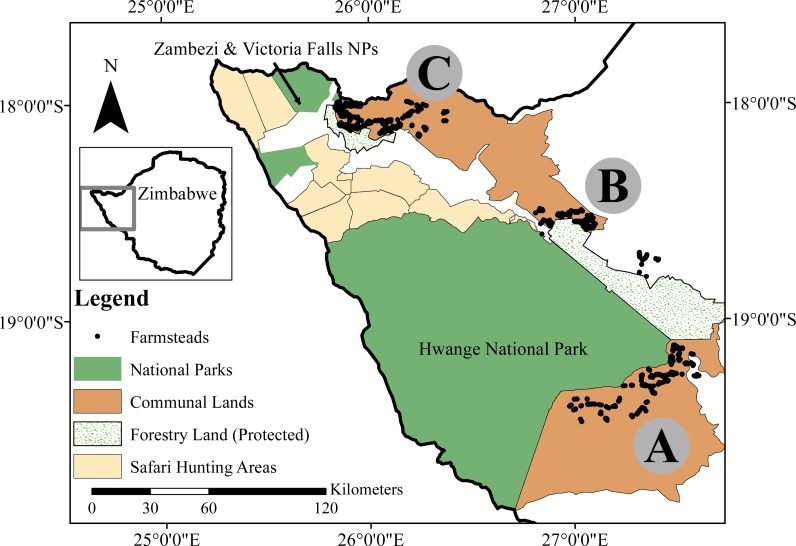
Our study area in northwestern Zimbabwe, showing the locations of our three focus sites: the (A) Tsholotsho, (B) Mabale and (C) Mvuthu-Shana study sites. The various land-use categories are shown, and the rural farmstead dots represent a subset of all farmsteads in each site. Map created by Timothy Kuiper.

Our study area encompassed three separate rural communal land sites adjacent to National Parks (NPs) in northwestern Zimbabwe (Fig. 1). Two bordered on Hwange NP (HNP): Tsholotsho communal land (Matapula Chieftainship, 2,171 km^2^) and Mabale communal land (Dingani Chieftainship, 480 km^2^), while the third site, Mvuthu-Shana communal land (Mvuthu-Shana and Shana Chieftainships, 655 km^2^), was adjacent to Zambezi and Victoria Falls NPs (ZNP/VFNP). All three study sites and the NPs mentioned fall within the broader Kavango Zambezi Transfrontier Conservation Area which connects protected areas across northern Zimbabwe, Botswana, and Namibia, with those southern Zambia and Angola (http://www.kavangozambezi.org/). The study locations are hereafter referred to as the Tsholotsho, Mabale and Mvuthu-Shana study sites. The density of farmsteads (a farm and its buildings, typically with 3–4 rural huts) was 1.8/km^2^ in Tsholotsho, 6.2/km^2^ in Mabale and 6.1/km^2^ in Mvuthu-Shana. Ethnicity differed among study sites, with people living in Tsholotsho primarily speaking Ndebele (99%) as a first language, and people in Mabale and Mvuthu-Shana speaking a mix of Ndebele (48% and 57% respectively), Nambya (32% and 29% respectively), and numerous other regional African languages (20% and 14% respectively). The area has low-fertility soils (Kalahari sands) and low and erratic rainfall (inter-annual CV of 25%), with an annual average range of 540–630 mm (lowest in Tsholotsho and highest in Mvuthu-Shana, falling in November–March). Subsistence agro-pastoral farming practices are the dominant livelihood activity, with livestock kept for milk and meat production, ploughing, sale and barter.

Large predators largely remained within protected areas and we found no evidence to suggest any were permanently resident in communal lands. In the protected areas lions occurred at population densities of between 2.5 and 4.5/100 km^2^ (Loveridge et al., 2016), leopards at 1.46/100 km^2^ in the northeastern part of HNP (unpublished camera trap data) and hyaenas at 9.0/100 km^2^ (Periquet, 2014). Brown hyaenas (*Hyaena brunnea*), African wild dogs and cheetahs were present but relatively rare in the Hwange ecosystem (A Loveridge, pers. obs., 2016). Caracal (*Felis caracal*) and black-backed jackals (*Canis mesomelas*) are widely distributed in the ecosystem (A Loveridge, pers. obs., 2016).

### Monitoring carnivore attacks on livestock

Between January 2009 and December 2013, we carried out comprehensive monitoring of large carnivore attacks on livestock (predominantly cattle *Bos indicus*, donkeys *Equus asinus*, goats *Capra hircus* and sheep *Ovis aries*) in the three study sites. We defined an ‘attack’ as an event in which a large carnivore killed or injured one or more livestock individual(s). Data on attacks were collected by field research staff through a network of contacts in the community, including a ‘conflict hotline’ set up to solicit reports of attacks, inspection of official attack records, and regular meetings with village heads (who compile the official attack records within their administrative areas). Attack records from these sources were independently verified by field research staff who visited individual attack sites and collated a report for each predator attack as soon as possible after it occurred (modal time between attack and site visit = 1 day). The majority of incidents were those directly reported to the research project. Where possible, reports included the date and GPS coordinates of each incident, confirmed the predator species responsible for the attack (through examination of field signs, predator-specific injuries to livestock and sightings and sign of predators recorded by farmers), the species, number, and age (defined as juvenile <1 year, sub-adult 1–2 years and adult >2 years) of livestock lost, whether the incident occurred during the day or at night, whether the animals lost were wearing bells, and whether the incident occurred within a boma (protective livestock enclosure) or outside the protection of a boma. The average number of each livestock species kept (Table S2 ) and the proportion of livestock fitted with bells (Table S3) was assessed at 624 randomly selected rural farmsteads (representing around 10% of all farmsteads).

### Assessing predator-specific preference for different livestock species

We hypothesized that different carnivores would prefer to attack different livestock species. To test this for lions, hyaenas and leopards (the most common predators), we estimated prey preference for cattle, donkeys and goats (the most commonly attacked species) using Jacobs’ Index: *D* = (*r* − *p*)∕(*r* + *p* − 2*rp*) (Jacobs, 1974). Here *r* represented the proportion of attacks on each of the common livestock species for a particular predator and *p* represented the relative availability of each of these livestock species. Availability was determined from farmstead-level data on the relative abundance of each livestock species at each site (Table S2). Values of *D* range from −1 (maximum avoidance) to 1 (maximum preference). Analyses were carried out separately for each site.

### Livestock bells and vulnerability to depredation

We hypothesized that bells fitted to grazing livestock (as is common in southern Africa) might aid carnivores in locating and attacking unattended, grazing livestock. We therefore predicted that carnivores might selectively attack livestock with bells due to a learned association between the sound of bells and presence of livestock. To test this for lions and hyaenas, we estimated prey preference for livestock with and without bells using Jacobs’ Index (Jacobs, 1974). Here *r* represented the proportion of attacks on livestock animals with bells and *p* represented the relative availability of livestock animals with bells. Availability was determined as the proportion of the total livestock in each site with bells. Analyses were carried out separately for each site.

### Patterns of conflict in relation to bomas and time of day

We hypothesized that attacks on animals within bomas would occur infrequently given the protection they afford to livestock. For each site, we determined the proportion of attacks occurring inside and outside a boma for each predator and livestock species combination. Fisher’s exact tests were used to test for differences among predators and livestock species in the proportion of attacks occurring inside and outside bomas. We conservatively used a null hypothesis of equal time spent by livestock inside and outside bomas. Extensive discussions with local herdsman revealed that it is the norm in our study sites for all livestock species to spend most of the 24 h cycle within bomas; as a rule all species spend the night inside the boma and then spend a portion of daylight hours outside the boma grazing (L Sibanda, pers. comm., 2016). There are occasions when livestock are left out at night, but this is not common. Kuiper et al. (2015) show for example that cattle in Tsholotsho spend 83% of nights within bomas.

For 1,409 attacks, accurate data were available on the location of the incident as well as the ‘home’ farmstead to which the attacked animal belonged. To better understand the circumstances of attacks occurring outside bomas, we calculated the proportion of all attacks occurring 0–100 m, 100–1,000 m, and >1,000 m from the ‘home’ farmstead (the boma is present at the farmstead). We used data from all predator species, livestock species and study sites.

These analyses were repeated for the proportion of attacks occurring during the day and during the night.

### Seasonal patterns in attacks on livestock

We examined seasonal trends in attacks on livestock by testing for among-month differences in attack frequency between 2009 and 2013. We examined seasonality of attacks only for the most frequent predator species (lions and hyenas) and livestock species (cattle, donkeys and goats). Previous work has suggested the importance of seasonal cattle movements in explaining seasonal depredation by lions in the Tsholotsho study site (Kuiper et al., 2015). We tested whether seasonality in attacks on cattle was a general pattern across all study sites and whether this pattern held for other livestock species, so data for attacks on donkeys and goats were pooled as ‘other’ species, for comparison with attacks on cattle. Pilot analysis showed no seasonal patterns in the Mvuthu-Shana site. We pooled seasonal data from Tsholotsho and Mabale, as subsistence agricultural practices were similar (Kuiper et al., 2015) in both sites and, being on the boundary of HNP, both were likely to experience similar seasonal effects. Thus, our results provide a general picture of seasonality in livestock attacks in communal lands adjacent to HNP.

We tested for seasonality in (1) lion attacks on cattle, (2) lion attacks on ‘other’ species, (3) hyaena attacks on cattle, and (4) hyaena attacks on ‘other’ species. Since our response variable was a non-normal count (the number of attacks recorded in a particular month) and the analysis included a random effect, we performed analysis using generalized linear mixed models (GLMM) (Zuur et al., 2009). Count data were over-dispersed (variance larger than the mean), so we used a negative binomial error structure for the GLMMs (Ver Hoef & Boveng, 2007; Zuur et al., 2009). We carried out four separate GLMMs for each of (1)–(4) above. For each model, the total number of attacks recorded in a particular month was treated as a data point, so each model included 60 observations (12 months × 5 years). We used month as the fixed effect in each model, and included year as a random effect in each model to account for among-year variation in attacks and to allow generalization of trends across years. The significance of the month fixed effect was assessed using likelihood ratio tests of the full model including the effect and the reduced model excluding the effect (Zuur et al., 2009). We used the R package *glmmADMB* to run the mixed models (Skaug et al., 2011).

### Incident reporting, financial losses and boma assessments

We recorded whether attacks had been officially reported and whether to local authorities (village head, local police, hunters or local chief) or to wildlife authorities (Rural District Council, RDC or the Parks and Wildlife Management Authority, PWMA). Local market values for each species of livestock were used to estimate overall financial losses experienced for each site. Protective livestock enclosure (boma) construction was assessed using the following measures: material used (poles, brush, diamond mesh or barbed wire), wall height (cm) and visibility through the boma wall. Visibility through the boma wall was quantified by recording the proportion of squares visible on checkerboard (with 100, 2 × 2 cm squares), held inside the boma wall, one meter above the ground, every 10 m (visibility scores were averaged for each boma).

This study did not include human participants or record any personal data. The study did not make use of captive or domestic research animals, nor capture or experiment on wild animals. Field permits were in place for the research from the Zimbabwe Parks and Wildlife Management Authority (Permit numbers: REF: DM/Gen/(T) 23(1)(c)(ii): 03/2008, 03/2009, 25/2010, 06/2011, 12/2012, 08/2013, 51/2014). Permission to undertake this work was given by the Hwange and Tsholotsho Rural District Councils and by local traditional leaders.

## Results

### General patterns

A total of 1,527 separate predator attacks on livestock were recorded between January 2009 and December 2013. A total of 2,039 head of livestock was killed, while a further 306 were injured (Table S1). Of the total attacks, 49% were recorded in Tsholotsho (*n* = 744), 31% in Mabale (*n* = 479), and 20% in Mvuthu-Shana (*n* = 304). Most attacks (93%, *n* = 1, 425) involved death to livestock with a mean of 1.47 (±1.19 (SD), range: 1–17) head of livestock killed per fatal incident, while the remainder (*n* = 102) involved only injury. Nine percent (*n* = 133) of fatal attacks involved injury to additional domestic animals. Over the study period, we calculated average yearly losses of 0.047 (±0.026 (SD)) head of livestock per farmstead in Tsholotsho, 0.045 (±0.023) in Mabale, and 0.022 (±0.008) in Mvuthu-Shana. Overall, 12.6% of attacks occurred on livestock grazing in forestry lands, with the remainder occurring on communal land. In Mabale, 37.4% of attacks occurred in protected forest land (3% in Tsholotsho, 11.9% Mvuthu-Shana).

**Figure 2:**
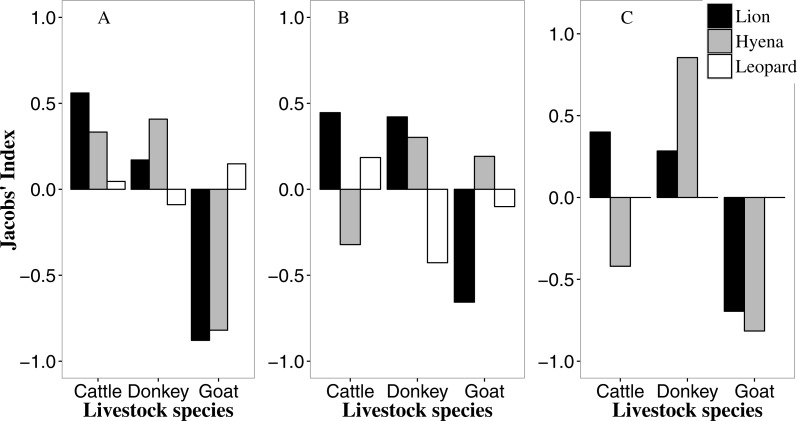
Predator-specific preference (Jacobs’ index) for different livestock species in the Tsholotsho (left), Mabale (centre) and Mvuthu-Shana (right) study sites (2009–2013). We did not determine selection for leopards in Mvuthu-Shana as there were only four attacks involving leopard (Table S1).

The number of livestock killed per attack differed between predator species (Kruskal–Wallis test: ${\chi }_{\mathrm{(2)}}^{\mathrm{2}}=26.17$, *P* < 0.01) with two or more animals killed per attack on 28.1% (*n* = 238), 42.1% (*n* = 24) and 18.5% (*n* = 124) of occasions by lions, leopards and hyaenas, respectively. Lions and hyaenas attacked livestock most frequently, with a small number of attacks by leopards and very few attacks by cheetah, caracal and black-backed jackal (Table S1). In all three sites the proportion of attacks by lions, hyaenas and leopards were significantly different from equal (Table S1; Tsholotsho: ${\chi }_{\mathrm{(2)}}^{\mathrm{2}}=350.86$, *P* < 0.01; Mabale: ${\chi }_{\mathrm{(2)}}^{\mathrm{2}}=184.77$, *P* < 0.01; Mvuthu-Shana: ${\chi }_{\mathrm{(2)}}^{\mathrm{2}}=163.50$, *P* < 0.01). Leopards were responsible for fewer than 10% of attacks in each site. Hyaenas were responsible for most attacks in Tsholotsho (58%), while lions were responsible for most attacks in Mabale (57%) and Mvuthu-Shana (61%). The proportion that lion attacks made up of all incidents at each site was not significantly different among sites (${\chi }_{\mathrm{(2)}}^{\mathrm{2}}=5.25$, *P* = 0.072). The same was true for leopard attacks (${\chi }_{\mathrm{(2)}}^{\mathrm{2}}=4.28$, *P* = 0.117), while the proportion that hyaena attacks made up all incidents was significantly different among sites (${\chi }_{\mathrm{(2)}}^{\mathrm{2}}=5.25$, *P* = 0.020).

Cattle were the most frequently attacked livestock species, followed by donkeys and goats. There were very few recorded attacks on other livestock species. The number of attacks on cattle, donkeys and goats were significantly different from equal in all three sites (Table S1; Tsholotsho: ${\chi }_{\mathrm{(2)}}^{\mathrm{2}}=346.85$, *P* < 0.01; Mabale: ${\chi }_{\mathrm{(2)}}^{\mathrm{2}}=101.28$, *P* < 0.01; Mvuthu-Shana: ${\chi }_{\mathrm{(2)}}^{\mathrm{2}}=75.13$, *P* < 0.01). In Tsholotsho and Mvuthu-Shana donkeys were attacked more frequently than goats, while in Mabale goats were attacked more frequently than donkeys. Farmsteads in the three sites owned similar numbers of cattle and goats, but dissimilar numbers of donkeys, with fewest donkeys owned in Mabale (Table S2).

### Assessing predator-specific selection for different livestock species

In all sites lions selectively attacked cattle and donkeys (with strongest selection for cattle), but avoided goats (Fig. 2, and see this figure for all livestock preference results discussed below). Most livestock kills by lions (58.1%) were adult cattle and donkeys (Fig. S1; see this figure for all age preference results discussed below). Hyaenas showed strongest selection for donkeys in all sites, while avoiding goats in Tsholotsho and Mvuthu-Shana, but showing weak selection for goats in Mabale. Hyaenas avoided attacking cattle in Mabale and Mvuthu-Shana, but showed some selection for cattle in Tsholotsho. As with lions, most hyaena kills (59.7%) were adult cattle and donkeys. In Tsholotsho, leopards attacked cattle and donkeys in proportion to their availability, while showing weak selection for goats. In Mabale, leopards selected for cattle, avoided donkeys and attacked goats in proportion to their availability. Overall, leopards only rarely killed donkeys (3.6% of kills) and rarely killed adult cattle (of leopard cattle kills, 43.3% were yearlings and 50.0% were calves). Of leopard kills, 55.4% were goats, of which 78.2% were adult.

### Livestock bells and vulnerability to depredation

**Figure 3:**
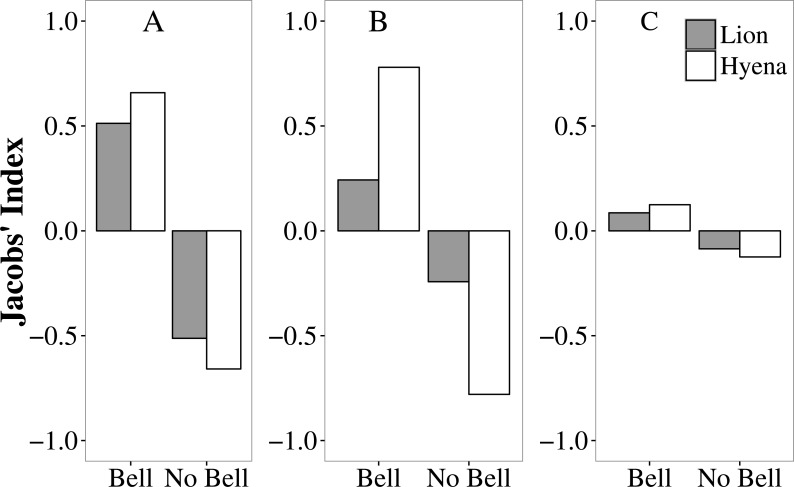
Selection (Jacobs’ Index) by lions (grey bars) and hyaenas (white bars) for livestock with and without bells in the Tsholotsho, Mabale and Mvuthu-Shana study sites (2009–2013). More positive values indicate preference while more negative values indicate avoidance.

In Tsholotsho and Mabale, lions and hyaenas preferentially selected livestock wearing bells, attacking them more frequently than would be expected from the proportion of the total number of livestock with bells in each site (Table S3; Fig. 3). In both Tsholotsho and Mabale, hyaenas showed even stronger selection for animals with bells than did lions. In Mvuthu-Shana, both lions and hyenas attacked livestock with bells in approximate proportion to their availability, only weakly selecting livestock with bells.

### Patterns of conflict in relation to bomas and time of day

**Figure 4:**
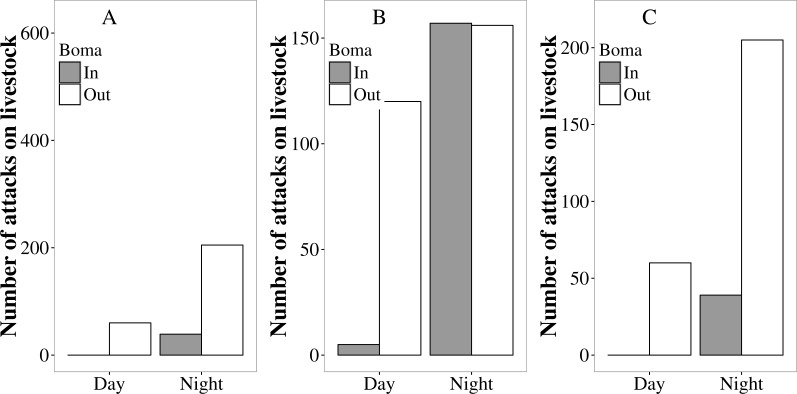
The number of attack incidents on livestock occurring inside (grey bars) and outside (white bars) protective bomas during the day and the night in Tsholotsho, Mabale and Mvuthu-Shana communal lands (2009–2013).

Overall, more attacks on livestock occurred at night than during the day (80% vs. 20%; ${\chi }_{\mathrm{(1)}}^{\mathrm{2}}=540.53$, *P* < 0.01; Fig. 4), and significantly more livestock were attacked outside the protection of a boma than were attacked whilst inside (83% vs. 17%; ${\chi }_{\mathrm{(1)}}^{\mathrm{2}}=657.78$, *P* < 0.01; Fig. 4). The majority (64%) of attacks occurred at night and outside a boma. These patterns were generally consistent among sites, although in Mabale significant numbers of livestock were attacked while inside bomas at night (Fig. 4). The majority of both day-time and night-time attacks on livestock occurred at locations further than 1 km from the home boma, with a small minority of attacks occurring outside but within 100 m from the home boma (Table 1). In all sites, the proportion of attacks occurring at night was significantly different from equal among lions, hyenas and leopards (Fisher’s exact test *P* < 0.05; Fig. S2). Similarly, in all three sites the proportion of attacks on cattle, goats and donkeys occurring at night was significantly different from equal (Fisher’s exact test *P* < 0.05). Hyaenas were the most nocturnal in their attacks on livestock, with more than 90% of attacks on all livestock species in all sites occurring at night, excepting the case of attacks on cattle in Mabale (87% at night). In all sites and for all livestock species, the proportion of lion attacks occurring during the night was lower than for hyenas (Fig. S2). For both lions and hyaenas, a higher proportion of the attacks on cattle occurred during the day than was the case for attacks on donkeys and goats. This difference was clearest for lion attacks, with around 40% of lion attacks on cattle occurring during the day in all sites. Across all sites and livestock species, there was no day/night pattern for leopard attacks.

**Table 1:** The number of attacks occurring at different distances from the home boma of the attacked individual, stratified by study site and time of day.

Location	Time	Total incidents	No. of attacks at different distances from boma			
			Inside	<100 m	100 m–1 km	>1 km
Tsholotsho	Day	94	1 (1%)	1 (1%)	16 (17%)	76 (81%)
	Night	601	42 (7%)	20 (3%)	140 (23%)	399 (66%)
Mabale	Day	115	5 (4%)	4 (3%)	28 (24%)	78 (68%)
	Night	298	157 (53%)	28 (9%)	36 (12%)	77 (26%)
Mvuthu-Shana	Day	59	0 (0%)	5 (8%)	13 (22%)	41 (69%)
	Night	242	39 (16%)	14 (6%)	83 (34%)	106 (44%)

A higher proportion of attacks occurred inside bomas in Mabale compared to Tsholotsho and Mvuthu-Shana (Fig. 4; Fig. S3). In Mabale and Mvuthu-Shana, but not Tsholotsho, the proportion of attacks occurring outside a boma was significantly different from equal among lions, hyaenas and leopards (Fig. S3; Fisher’s exact test, Tsholotsho: *P* = 0.085, Mabale: *P* = 0.001, Mvuthu-Shana: *P* = 0.001. In all three sites the proportion of attacks on cattle, goats and donkeys occurring outside a boma were significantly different from equal (Fig. S3; Fisher’s exact test *P* < 0.05). For all sites and predators, a considerably higher proportion of attacks occurred inside a boma for goats than for cattle and donkeys, excepting hyaena attacks on goats in Mvuthu-Shana (Fig. S3). In all sites and for all livestock species, the proportion of lion attacks occurring inside a boma was higher than for hyaenas (Fig. S3).

### Seasonal patterns in attacks on livestock

**Figure 5:**
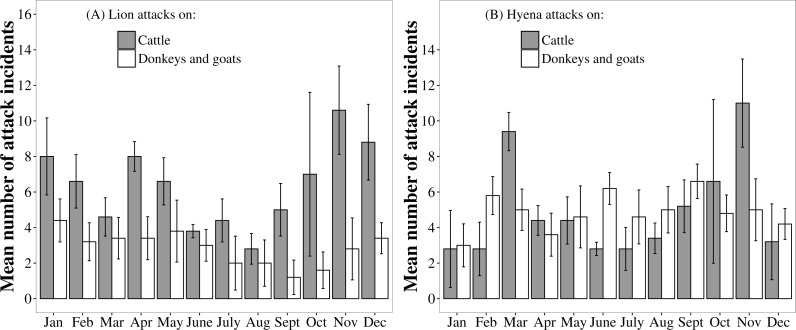
Monthly patterns in the frequency of lion and hyaena attacks on different livestock species. Data are pooled from the Tsholotsho and Mabale study sites (2009–2013). Bars represent monthly means across the five years and error bars represent standard error.

Month had a significant effect on the frequency of lion attacks on cattle (Likelihood ratio test for GLMM: ${\chi }_{\mathrm{(11)}}^{\mathrm{2}}=20.45$, *P* = 0.040), suggesting that attack frequency was seasonal. Lion attacks on cattle were least frequent during the dry season months of June to September and were most frequent during the wet season months of November to January (Fig. 5).

By contrast, the frequency of lion attacks on donkeys and goats was not significantly different among months (${\chi }_{\mathrm{(11)}}^{\mathrm{2}}=16.05$, *P* = 0.140). The frequency of hyaena attacks on cattle was significantly different among months (${\chi }_{\mathrm{(11)}}^{\mathrm{2}}=29.45$, *P* = 0.002), with attack frequency peaks in November and March. The frequency of hyaena attacks on donkeys and goats was not significantly different among months (${\chi }_{\mathrm{(11)}}^{\mathrm{2}}=4.77$, *P* = 0.940).

### Incident reporting, financial losses and boma assessments

Of the 2,054 reports made of 1,089 lion conflict incidents (reports of the same incident were made to multiple agencies), only 12.5% were made to the RDC) with 5.9% made to the PWMA, 31.5% were made at local level (village heads, local police, hunters or local chief) and 15.4% were unreported (the latter were recorded by field research staff only). For hyaenas, of 1,425 reports made of 811 incidents, only 2.5% were made to RDC and only 0.4% to PWMA (38.7% were made locally and 17.2% unreported). Thus, while similar proportions of lion and hyaena attacks were reported to local or village authorities, a much lower proportion of hyaena attacks were reported to wildlife managers. It is noteworthy that in Tsholotsho, where hyaenas were responsible for more conflict incidents and caused similar levels of financial damage to lions, the same trend in underreporting was apparent. For lion depredation events, 13% of reports were to RDC authorities and 4% to PWMA, for hyaena only 3% of reports were to the RDC and none were to PWMA, suggesting that even in an area where depredation rates by hyaenas was high, damage was consistently underreported at higher management levels compared to lion depredation. Project and RDC records show that around HNP, between 2000 and 2013, 32 lions were officially destroyed on problem animal control (PAC) licences, while only one hyaena was destroyed over the same period. Similarly, in the Mvuthu-Shana site (Hwange RDC West and Central) 13 lions and no hyaenas were destroyed as problem animals between 2003 and 2013.

Based on the compensation value of livestock, the average annual (2009–2013) financial loss to predators across the three study sites was US$49 412, $28 510, $1 347 and $79 for lions, hyaenas, leopard and cheetahs, respectively (Table S4). Lions tended to kill more and higher value livestock species (cattle and donkeys), resulting in greater financial losses to lions than to hyaenas or leopards. The exception to this was in Tsholotsho where a higher proportion of kills were made by hyaenas compared to lions resulting in very similar financial impacts for the two species (lions: $17,029, hyaenas: $17,345). Total annual financial losses to all large predators were highest in Tsholotsho ($34,882 average per year) and lowest in Mabale ($13,054 average per year). However, whilst the Tsholotsho study site was 4.5 times as large as the Mabale site, Mabale had around 30% more farmsteads. When corrected for the number of farmsteads, Tsholotsho still had the highest losses ($8.93 per farmstead), followed by Mvuthu-Shana ($7.79 per farmstead) and Mabale ($4.34).

Of 624 farmsteads assessed, 507 farmsteads (Mabale 108, Tsholotsho 191 and Mvuthu-Shana 208) had a boma in which to keep their livestock (114 farmsteads did not keep livestock and three kept livestock but had no boma). In Tsholotsho, 85% of bomas assessed were constructed of sturdy timber poles compared to only 29 and 32% in Mabale and Mvuthu-Shana (where the majority of bomas were constructed primarily of steel fencing wire or wire mesh). Consequently, visibility scores were significantly lower in Tsholotsho (mean percentage visibility scores (±SD) differed between sites: Mabale 68.9% ± 30.9, Tsholotsho 37.3% ±20.9, Mvuthu-Shana 65.5 ± 29.3, Kruskal–Wallis test: ${X}_{(2)}^{2}=$ 105.81, *P* < 0.01). Boma walls were higher in Tsholotsho than those in Mabale or Mvuthu-Shana (height in centimetres (±SD), Mabale =102 cm ± 41.1; Tsholotsho 123.5 ± 34.9, Mvuthu-Shana 113.8 ± 29.4, Kruskal–Wallis test: ${X}_{(2)}^{2}=32$.17, *P* < 0.01). In Tsholotsho, 24% of livestock owners kept guard dogs at their farmsteads compared to 13.8% and 5.7% in Mabale and Mvuthu-Shana, respectively.

## Discussion

Our analysis illustrates that conflict between people and predators is multifaceted and complex, pointing to the interconnected roles of predator behaviour and husbandry practices in determining where, why and how intensely conflict arises. Among our study sites, we recorded both differences and similarities in predator attacks on livestock and the effects of husbandry on such attacks. We discuss each of our results in turn below, referring in each subsection to both the role of predator behaviour and husbandry practices in explaining the observed patterns.

### General patterns

Lions and hyaenas were predominantly responsible for attack incidents. There was variation in frequency of attacks by different predator species among sites; hyaena attacks were more frequent than lion or leopard in Tsholotsho, but lion attacks were most frequent in Mabale and Mvuthu-Shana. Reasons for these differences are difficult to disentangle and could be due to variations in livestock species kept, husbandry practices, varying density of human settlements, or variation in predator densities. The higher proportion of hyaena attacks in Tsholotsho, for example, may be explained by their observed preference for donkeys across all sites and the higher abundance of donkeys at Tsholotsho compared to other sites. Cattle were the most frequently attacked species, likely because they were also the most commonly kept livestock and preferred by lions and hyaenas, the predominant predators. Leopard attacks were considerably less frequent than attacks by lions and hyaenas and were more variable in both time and location, reflecting the well described opportunism of this predator (Kingdon & Hoffmann, 2013). The large felids appear more likely to make multiple kills in a single predation event (this is particularly the case for leopards where 42% of kills were of more than one animal. This may be because of their large size relative to preferred prey, which likely reduces the time needed to subdue prey and allows for further attacks (Kruuk, 1972). Lions are more likely to hunt with conspecifics, and the multiple kills observed may also be the result of attacks by more than one lion.

### Assessing predator-specific selection for different livestock species

Studies that assume different species of large predator behave in a similar way are likely to underestimate the complexity, adaptability and variability of predator behaviour. Predators forage so as to maximise food intake while limiting energy expenditure and risk of injury during hunts (Pyke, 1984). In wild ecosystems, large carnivores of different body sizes show a preference for specific size ranges of prey (Hayward, 2006; Hayward et al., 2006; Hayward & Kerley, 2005). Lions prefer prey ranging from 190–550 kg (mode 290 kg), hyaenas prefer prey of 56–182 kg (mode 102 kg) and leopards, prey of 10–40 kg (mode 25 kg). In our study, lions preferred cattle (mean weight African/ European crossbreed 312 kg; weight range 175–450 kg, livestock weights from Wint, Slingenbergh & Rogers (2000)), but also selected donkeys (125 kg). Hyaenas selected donkeys at all sites and, while leopards did not show a marked preference for any domestic species, they primarily killed goats (25 kg) and calves. Similar patterns were apparent in ages of livestock of different species killed. Lions killed mostly adult cattle and donkeys, hyaenas killed mostly adult donkeys and sub-adult cattle, and leopards killed goats of all ages and cattle calves. In all cases, preferred domestic stock body sizes closely approximated modal sizes of preferred wild prey suggesting that prey body size preference extends to depredation on domestic stock.

### Livestock bells and vulnerability to predation

Analysis of movement patterns of lions show that stock raiders often purposefully approach protective enclosures at night to assess predation opportunities (Oriol-Cotterill et al., 2015; Valeix et al., 2012). This reflects the overall pattern of active hunting in many large carnivore species, where prey items are actively sought out (Kingdon & Hoffmann, 2013). Similarly, active and potentially habitual hunting behaviour was observed in our study where domestic animals fitted with bells were more likely to be selected by predators than those without. Both lions and hyaenas showed this preference and appear to have developed a learned association between the sound of bells and the presence of livestock. Bells therefore appear to increase livestock vulnerability to predation (by providing an auditory cue to predators. Simply removing bells, however, is not a practical solution. Bells may reduce livestock losses by making it easier for herders to attend grazing cattle and to find them for rounding up at night. Our results may potentially have been biased by the possibly more common use of bells by farmsteads in areas where predator encounters are frequent or where livestock are less closely attended (factors increasing attack frequency). In our Hwange NP study area, it was found that simply ringing a cow bell from a vehicle attracts lions (A Loveridge, pers. obs., 2016), suggesting it is the sound rather than other cues that attract the predators. If the learned association between the sound of bells and availability of livestock influences predatory behaviour, a learned aversion might also be induced by linking distasteful or negative experiences with cues that trigger predatory behaviour. This may be a mitigation strategy that warrants further attention.

### Livestock predation in relation to bomas and time of day

One widely recommended requirement in many human-predator conflict situations is improved protection of livestock. Improved husbandry practices invariably reduce predator attacks by limiting the accessibility of livestock to predators (Linnell et al., 1996; Ogada et al., 2003). For example, in Nepal, snow leopard (*Panthera uncia*) predation is alleviated by well-designed, predator-proof enclosures in which to keep stock at night (Jackson, Wangchuk & Hillard, 2002). In Africa, protective bomas have traditionally been used to protect livestock, and recent innovations to strengthen these have reduced losses even further (Lichtenfeld, Trout & Kisimir, 2015; Ogada et al., 2003).

In this study, the majority of livestock were lost at night when roaming outside of and away from protective bomas. Sixty four percent of all attacks occurred when livestock were left outside bomas at night. The small proportion of attacks occurring within bomas is significant when one considers that livestock individuals in our study sites spend most of their time within bomas, leaving only during daylight hours to graze (L Sibanda, pers. obs., 2016) and are only occasionally left out at night (Kuiper et al., 2015) . In our study sites, more assiduous use of protective enclosures at night could greatly reduce the availability of livestock to predators and hence reduce overall losses. A recent study of cattle movements in the Tsholotsho site (Kuiper et al., 2015) found that on average cattle spend 62 nights a year outside the protection of bomas. This accords with a study in Botswana (Valeix et al., 2012) in which the majority of livestock killed were stray animals left grazing far from protective enclosures at night. By contrast, studies in Kenya (Woodroffe et al., 2007) and Tanzania (Kissui, 2008) showed that livestock are generally well tended when grazing, and most livestock killed were taken from within bomas (Kolowski & Holekamp, 2006).

Most large African predators, apart from cheetah, are crepuscular or nocturnal, but all show a high degree of flexibility and opportunism, particularly in human disturbed environments (Oriol-Cotterill et al., 2015; Valeix et al., 2012). Differing activity periods are behavioural adaptations to maximise foraging opportunities, limit competitive interactions, avoid predation or avoid periods when humans are active (Harmsen et al., 2009; Oriol-Cotterill et al., 2015). Compared to hyaenas, lions and leopards were more likely to attack livestock during the day (usually killing vulnerable cattle or goats in grazing areas), although most attacks by these species still occurred at night. Lion attacks on cattle occurred more frequently during the day than other predator attacks or lion attacks on other livestock species. A recent analysis suggests that lion attacks occurred when cattle were left to graze unattended during the day in close proximity to habitat lions used (Kuiper et al., 2015). This finding accords with our results which show that 40% of lion attacks on cattle occur during the day (this is when cattle are typically out grazing and attacks are unlikely to occur if cattle are attended). This highlights the need for day-time herd guarding strategies to reduce predation on grazing cattle by lions.

Hyaena kills were almost exclusively at night, reflecting their predominantly nocturnal activity patterns. This might also be explained by a high degree of opportunism by hyaenas in approaching human settlements at times when people are least active and taking advantage of livestock left outside bomas at night. Similar patterns of attacks on livestock by these predator species were reported in Tanzania (Kissui, 2008). Our data suggest that hyaena attacks can be almost entirely mitigated by keeping stock in strong bomas at night. In contrast, lion and leopard attacks require both strong night-time bomas and daytime supervision of grazing livestock. The large felids were more likely to attack stock in bomas than were hyaenas, likely because felids are highly adapted to jumping and climbing, which aids entry into a high walled enclosures (Kolowski & Holekamp, 2006). In areas where such entry is problematic, roofs made of diamond mesh can be used (Jackson, Wangchuk & Hillard, 2002).

Of those animals attacked when inside bomas (17% of incidents), most occurred at the Mabale study site. Although individual bomas were not assessed for each incident, our farmstead survey data suggest that bomas in Mabale were less robustly constructed than at the other sites, with many made of low barbed wire fences on wooden poles. Such structures are not predator proof and the high visibility through the boma walls likely meant that predators were more likely to attempt to enter or livestock to break out at the approach of a predator(with both scenarios increasing the likelihood of livestock loss). In addition, fewer farmsteads in this site kept guard dogs or donkeys, both species that are known to deter smaller predators and raise the alarm when larger predators are nearby (Marker, Dickman & Macdonald, 2005). By contrast, bomas in Tsholotsho tended to be constructed of sturdy timber poles, planted close together to form a high wall that largely obscured the livestock from view. Farmsteads in this site were more likely to own domestic dogs and keep donkeys (both of which raise the alarm at the approach of predators). These factors could explain why fewer attacks occurred in bomas at this site. In Mvuthu-Shana bomas were more robust than those in Mabale, but less well constructed than those in Tsholotsho, which is reflected in the intermediate levels of stock loss from bomas at this site. Availability of building materials may influence boma construction and therefore predation levels between sites. The Tsholotsho site is situated in heavily wooded teak (*Baikiaea plurijuga*) forest with high availability of timber for construction. In sites such as Mabale, where natural materials for boma construction are less readily available, other innovative solutions for livestock enclosures such as bomas with ‘living walls’ (Lichtenfeld, Trout & Kisimir, 2015) or mobile bomas constructed of PVC sheeting (which we are beginning to use in our Hwange study site) could be appropriate.

### Predator behaviour and spatial patterns of livestock attacks

Predators face a ‘landscape if fear’ in human-dominated areas, adjusting both their spatial movements and activity periods to avoid potentially fatal contact with people (Valeix et al., 2012). The location of livestock in relation to human settlement likely influences depredation rates as it is less of a risk for predators to attack livestock further away from human activity (Loveridge et al., 2016). This may explain why a significant number of livestock attacks across our study sites occurred at distances greater than 1km from the home boma. Similarly, the location of pasture lands in relation to wildlife sites and therefore the potential frequency of encounters with predators is also an important factor that may determine levels of predation on livestock. In this study, diurnal attacks on grazing animals were more common in the Mabale site. Significantly, many farmsteads in this site used the adjacent Sikumi Forest Land for grazing. Here, encounters with predators were much more likely than in community land and indeed many (37.4%) of the predation events recorded at this site occurred within the forest. Similarly, at the Tsholotsho site, cattle were more vulnerable to attack when they grazed closer to the Hwange National Park boundary than when kept close to settlements (Kuiper et al., 2015). Similar patterns in jaguar (*Panthera onca*) and cougar predation were seen in Brazil when cattle grazed in proximity to forest fragments (Mazzoli, Graipel & Dunstone, 2002; Palmeira et al., 2008), and in southern Africa livestock grazing close to protected areas were more vulnerable to attack by predators (Valeix et al., 2012). The effective day-time guarding of grazing livestock by people, guard dogs or donkeys and the selection of grazing areas distant from wildlife habitat are other strategies that reduce losses to predators (Kuiper et al., 2015; Marker, Dickman & Macdonald, 2005; Mizutani, 1997).

### Seasonal patterns in attacks on livestock

Seasonal patterns were apparent in attacks on cattle (but not other livestock species) by lions and hyaenas with higher livestock losses observed during the wet season. Similar seasonal patterns have been observed in other areas (Kissui, 2008; Patterson et al., 2004; Woodroffe & Frank, 2005). An explanation for this may be lower availability of wild prey during the wet months. In dry savannah ecosystems, ungulates prey aggregate predictably around water resources in the dry months (Davidson et al., 2012) and are more vulnerable to predation during this period due to lower body condition (Owen-Smith & Mills, 2008). Wild prey often disperse in the wet season to abundant high quality forage attain higher body condition, reducing vulnerability to predation. In some areas, this prompts predators to ‘switch’ to domestic animals (Valeix et al., 2012).

Seasonal variation in herding of cattle also plays a role in depredation seasonality (Kuiper et al., 2015). In the wet season, fields close to villages are planted with crops. To avoid crop damage, cattle are taken to graze in forested habitats within or in proximity to protected areas. In the dry season, after harvest, cattle graze close to villages and are fed on crop residues in fallow fields. Use of grazing close to villages as well as higher availability of wild prey may reduce vulnerability of cattle to predation in the dry season. Predation on cattle was seasonal, while predation on other domestic species was not, suggesting that seasonal herding may perhaps be more important than wild prey availability in determining depredation rates by carnivores. If wild prey availability was the dominant driver of depredation rates on domestic stock, one might expect depredation on all stock species to be seasonal. An alternative explanation for this is that donkeys, sheep and goats are not seasonally herded into vulnerable areas as is the case with cattle. Small stock (goats and sheep) are kept in close proximity to farmsteads year round, and donkeys tend not to be as vigilantly herded as cattle.

### Patterns of financial loss and conflict-incident reporting

The preference that lions showed for cattle at all three sites meant that they caused a disproportionate amount of financial damage (cattle are the most valuable livestock species). While hyaenas showed weak selection for cattle in Mabale and Mvuthu-Shana, they appeared to target them in Tsholotsho, killing more cattle than lions did. Total annual losses and annual per farmstead losses were highest in Tsholotsho, the site with highest levels of lion and hyaena depredation. Whilst estimates of per farmstead losses across entire communities are likely an unrealistic measure which under-represents individual losses (as not all farmsteads experience predation), it does allow comparison between sites. Losses per farmstead at the three study sites ($4.34–$8.93) were considerably lower than $40 per farmstead reported in Laikipia, Kenya (Woodroffe & Frank, 2005) and somewhat lower than $13 per farmstead reported in Gokwe, Zimbabwe (Butler, 2000). Additionally, the mean total annual losses of between $13,054 and $34,882 across our study sites, while high relative to per capita Gross National Income (GNI; $830; (World Bank, 2015), are low relative to the value of predator species, particularly lions. A single lion trophy hunt is valued at $100,000 (Lindsey et al., 2012), and individual lions have been valued at between $27,000–$500,000 to the photographic tourism industry (Thresher, 1981; Western & Henry, 1979). This suggests that if revenue streams derived from these species were used to benefit communities they would far outweigh losses to predation and provide significant incentives to tolerate large carnivores. In practice, local communities benefit very little from revenue derived from wildlife (Murombedzi, 1999). Hidden costs and negative perceptions towards conservation add to the financial losses (Kansky & Knight, 2014), creating critical impediments to conservation of large carnivores outside protected areas.

Our data on reporting patterns suggest that hyaena depredation is significantly under-reported to authorities responsible for wildlife management (RDC and PWMA), which may lead to biased problem-animal management policies and an under-appreciation of the damage caused by hyaena depredation. People may be more inclined to report lions as they are more conspicuous predators and pose a danger to human life, whereas hyaenas are ubiquitous, nocturnal and rarely injure people. Greater conservation attention is shown towards the large felids and, as a result, people may be more inclined to make reports knowing action will be taken. Additionally, lions are much easier to locate and destroy after depredation events than hyaenas, which are extremely wide ranging and difficult to locate. As a result, management agencies may be more likely to react to reports of problem lions than problem hyaenas. Furthermore, people have widely divergent perceptions of different carnivore species (Kellert et al., 1996). For instance, hyaenas are widely disliked by Kenyan farmers, despite causing lower financial loss than other carnivores (2001). In many African societies, spotted hyaenas have strong associations with witchcraft and the supernatural (Kingdon, 1977), which may also account for the ambivalence of local people towards depredation by this species.

### Conclusion

This study demonstrates the complex and related influences of predator ecology and human behaviour on livestock depredation. The differences among predators in the species of livestock preferred, diel and seasonal patterns of attack, and responses to husbandry practices (such as seasonal herding, the use of bomas, and the use of bells) highlight the importance of understanding predator behaviour when designing strategies to mitigate conflict. Our results also reveal that conflict intensity can be closely linked to husbandry practices, suggesting that changes in such practices should be at the centre of mitigation.

If large predators and agro-pastoralist people are to co-exist, it is essential that people benefit from predator presence and that the costs of conflict to local communities be mitigated. However, the success of solutions (such as better livestock protection) depends upon being able to predict important patterns of conflict (such as which livestock species are most vulnerable to which predator species) in order to correctly identify problems. Our results suggest that this is far from easy and, even within the limited geographical area covered by our three study sites, there was significant local variation in local human cultural and agricultural practices and the predatory behaviour and ecology of large carnivores. Solutions must take into account the complexity of biological and sociological systems at the interface of wild and human-dominated systems and at local scales. However, a common theme across virtually all predator-livestock conflict situations is that improved animal husbandry is likely to limit availability of livestock to predators. Our results indicate that, in our study sites, significant reductions in livestock losses can be achieved through adequate protection of livestock at night and supervision of grazing stock during the day. Finally, apparent patterns in predation on livestock may also be biased by differing levels of reporting for different predator species, perhaps influenced by attitudes towards different predators and perceptions of whether reports will be acted upon by management authorities.

##  Supplemental Information

10.7717/peerj.2898/supp-1 Supplemental Information 1 Supplementary materials

10.7717/peerj.2898/supp-2 Data S1 Raw data

## Additional Information and Declarations

### Field Study Permissions

The following information was supplied relating to field study approvals (i.e., approving body and any reference numbers): Field permits were in place for the research from the Zimbabwe Parks and Wildlife Management Authority (Permit numbers: REF:DM/Gen/(T) 23(1)(c)(ii): 03/2008, 03/2009, 25/2010, 06/2011, 12/2012, 08/2013, 51/2014). Permission to undertake this work was given by the Hwange and Tsholotsho Rural District Councils and by local traditional leaders.

### Data Availability

The following information was supplied regarding data availability: The raw data has been supplied as a Supplementary File.
