# Supplementary material for: Bells, bomas and beefsteak: complex patterns of human-predator conflict at the wildlife-agropastoral interface in Zimbabwe
Source: PeerJ. 2017 Jan 24;5:e2898. doi: 10.7717/peerj.2898 (PMC5267574; doi:10.7717/peerj.2898)
Supplement: Supplemental Information 1 [file peerj-05-2898-s001.docx]

ONLINE SUPPLEMENTARY MATERIAL

Loveridge et al, **Bells, bomas and beefsteak:** **Complex patterns of human-predator conflict at the wildlife-agropastoral interface in Zimbabwe**

Table S1. Number of attack incidents by predator and livestock species in (A) Tsholotsho, (B) Mabale and (C) Mvuthu study sites (2009 - 2013).

| Site | Predator | Livestock Species | | | | | | Total | % of total |
| --- | --- | --- | --- | --- | --- | --- | --- | --- | --- |
|  |  | Cattle | Donkey | Goat | Pig | Sheep | Dog |  |  |
| (A)Tsholotsho | Lion | 204 | 72 | 12 | 0 | 0 | 2 | 290 | 38.98 |
|  | Hyaena | 249 | 155 | 27 | 0 | 0 | 0 | 431 | 57.93 |
|  | Leopard | 9 | 2 | 10 | 0 | 0 | 0 | 21 | 2.82 |
|  | Cheetah | 1 | 0 | 0 | 0 | 0 | 0 | 1 | 0.13 |
|  | Caracal | 0 | 0 | 1 | 0 | 0 | 0 | 1 | 0.13 |
|  | Total | 463 | 229 | 50 | 0 | 0 | 2 | 744 | 100.00 |
|  | % of total | 62.23 | 30.78 | 6.72 | 0 | 0 | 0.27 | 100.00 |  |
|  |  |  |  |  |  |  |  |  |  |
| (B) Mabale | Lion | 179 | 44 | 44 | 4 | 2 | 3 | 276 | 57.26 |
|  | Hyaena | 46 | 21 | 94 | 1 | 5 | 0 | 167 | 34.65 |
|  | Leopard | 17 | 1 | 14 | 0 | 2 | 0 | 34 | 7.05 |
|  | Cheetah | 1 | 0 | 3 | 0 | 0 | 1 | 5 | 1.04 |
|  | Total | 243 | 66 | 155 | 5 | 9 | 4 | 482* | 100.00 |
|  | % of total | 50.41 | 13.69 | 32.16 | 1.04 | 1.87 | 0.83 | 100.00 |  |
|  |  |  |  |  |  |  |  |  |  |
| (C) Mvuthu | Lion | 117 | 41 | 19 | 6 | 1 | 1 | 185 | 60.66 |
|  | Hyaena | 28 | 75 | 7 | 0 | 0 | 0 | 110 | 36.07 |
|  | Leopard | 4 | 0 | 0 | 0 | 1 | 0 | 5 | 1.64 |
|  | Jackal | 0 | 0 | 5 | 0 | 0 | 0 | 5 | 1.64 |
|  | Total | 149 | 116 | 31 | 6 | 2 | 1 | 305* | 100.00 |
|  | % of total | 48.85 | 38.03 | 10.16 | 1.97 | 0.66 | 0.33 | 100.00 |  |

*Totals are greater than those quoted in the text because a small number of incidents involved injury and/or death to more than one livestock species.

Table S2. Average numbers of the three main livestock species per farmstead in the Tsholotsho, Mabale, and Mvuthu study sites.

| Study site | No. of farmsteads | Mean no. of livestock per household [mean$\boldsymbol{\pm}$SD (range)] | | |
| --- | --- | --- | --- | --- |
|  |  | Cattle | Donkeys | Goats |
| Tsholotsho | 232 | 4.68$\pm$6.32 (0-44) | 2.20$\pm$3.14 (0-16) | 4.64$\pm$6.05 (0-42) |
| Mabale | 121 | 4.24$\pm$5.60 (0-38) | 0.72$\pm$1.72 (0-10) | 4.72$\pm$4.61 (0-22) |
| Mvuthu | 271 | 3.83$\pm$5.36 (0-39) | 1.21$\pm$2.59 (0-20) | 3.37$\pm$5.46 (0-39) |

Table S3. A comparison between the proportion of livestock with bells in the Tsholotsho, Mabale and Mvuthu study sites and the proportion of livestock attacked by lions and hyaenas that had bells (2009-2013 attack incident data).

| Site | Proportion of livestock with bells | Proportion of attacked livestock with bells | |
| --- | --- | --- | --- |
|  |  | Lion | Hyaena |
| Tsholotsho | 0.165 (553 of 3342) | 0.381 (40 of 105) | 0.491 (105 of 214) |
| Mabale | 0.294 (396 of 1349) | 0.405 (15 of 37) | 0.770 (47 of 61) |
| Mvuthu | 0.234 (675 of 2884) | 0.266 (49 of 184) | 0.282 (31 of 110) |

Table S4. Mean annual financial losses (US dollar ± SD) incurred to the main predator species in each site (2009-2013), mean annual losses to all predators in each site (Total) and loss per farmstead in each site (annual loss per site/ number of farmsteads).

|  | Lion | Hyaena | Leopard | Cheetah | TOTAL | Total/ Household |
| --- | --- | --- | --- | --- | --- | --- |
| Tsholotsho | $17 029  ±6 388 | $17 345  ±10 135 | $472  ±326 | $35  ±79 | $34 882  ±14 931 | $8.93  ±382 |
| Mabale | $9 911  ±3 620 | $2 409  ±2 709 | $689  ±466 | $44  ±99 | $13 054  ±4 762 | $4.34  ±1.58 |
| Mvuthu | $22 472  ±18 671 | $8 755  ±5 557 | $185  ±338 | - | $31 413  ±22 315 | $7.79  ±5.53 |

Fig. S1. The age classes of domestic animals killed by predators expressed as a percentage of the total kills made by each predator (number of kills for which age class information available: lion: 1139, hyaena: 791, leopard: 83).


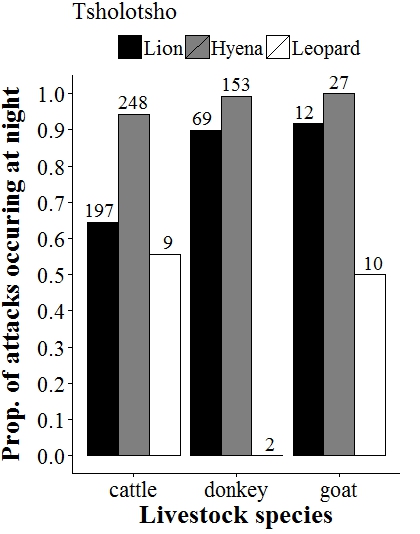

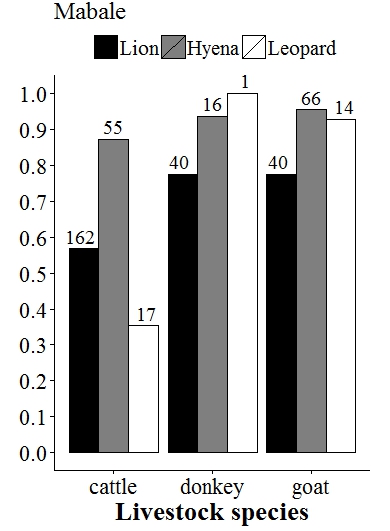

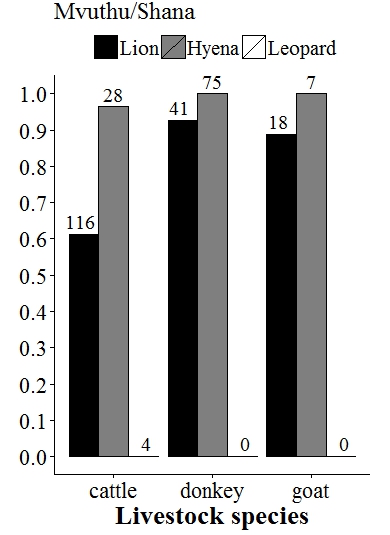


Fig. S2. The proportion of the total number of attacks for each predator and livestock species combination (e.g. lion attacks on cattle) that occurred during the night (2009 to 2013). The total number of attacks (day and night summed) for each predator/livestock species combination is shown above the relevant bar. Data for the Tsholotsho, Mabale and Mvuthu study sited are shown separately. Totals do not match totals in Table S1 identically as not all attacks had accurate information on the time of day of the incident.


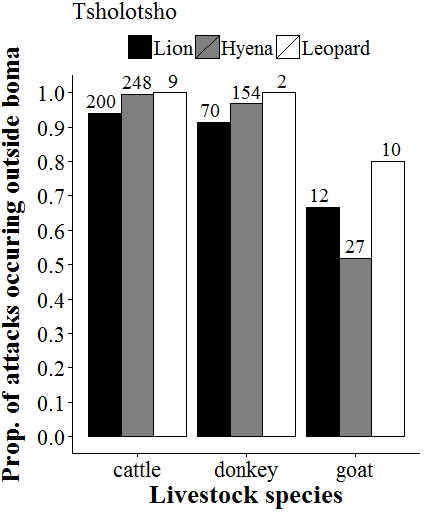

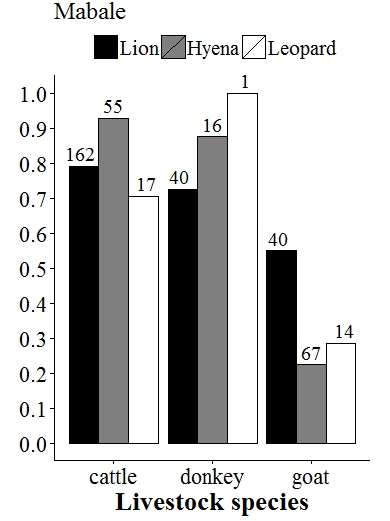

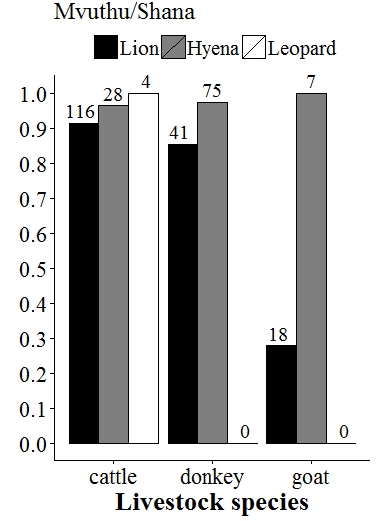


Fig. S3. The proportion of the total number of attacks for each predator and livestock species combination (e.g. lion attacks on cattle) that occurred outside the protection of a boma (2009 to 2013). The total number of attacks (inside and outside boma summed) for each predator/livestock species combination is shown above the relevant bar. Data for the Tsholotsho, Mabale and Mvuthu study sites are shown separately. Totals do not match totals in Table S1 because not all attacks had accurate information on whether or not the incident occurred inside a boma.
